# The Combined Use of Platelet-Rich Plasma and Adipose-Derived Mesenchymal Stem Cells Promotes Healing. A Review of Experimental Models and Future Perspectives

**DOI:** 10.3390/biom11101403

**Published:** 2021-09-24

**Authors:** Dimitris Tatsis, Varvara Vasalou, Efstathios Kotidis, Elissavet Anestiadou, Ioannis Grivas, Angeliki Cheva, Georgios Koliakos, Gregory Venetis, Manousos-George Pramateftakis, Nikolaos Ouzounidis, Stamatis Angelopoulos

**Affiliations:** 1 Fourth Surgical Department, School of Medicine, Aristotle University of Thessaloniki, 57010 Thessaloniki, Greece; vvasalou@gmail.com (V.V.); skotidis@gmail.com (E.K.); elissavetxatz@gmail.com (E.A.); mpramateftakis@hotmail.com (M.-G.P.); ouzoun21@yahoo.gr (N.O.); saggelopoulos@auth.gr (S.A.); 2 Oral and Maxillofacial Surgery Department, School of Dentistry, Aristotle University of Thessaloniki, 57010 Thessaloniki, Greece; gvenetis@dent.auth.gr; 3 Laboratory of Anatomy, Histology & Embryology, School of Veterinary Medicine, Aristotle University of Thessaloniki, 54124 Thessaloniki, Greece; janos@vet.auth.gr; 4 Department of Pathology, School of Medicine, Aristotle University of Thessaloniki, 54124 Thessaloniki, Greece; antacheva@yahoo.gr; 5 Department of Biochemistry, School of Medicine, Aristotle University of Thessaloniki, 54124 Thessaloniki, Greece; koliakos@med.auth.gr

**Keywords:** adipose-derived mesenchymal stem cells, platelet-rich plasma, wound healing, tissue regeneration, synergistic effect

## Abstract

Wound healing and tissue regeneration are a field of clinical medicine presenting high research interest, since various local and systematic factors can inhibit these processes and lead to an inferior result. New methods of healing enhancement constantly arise, which, however, require experimental validation before their establishment in everyday practice. Platelet-rich plasma (PRP) is a well-known autologous factor that promotes tissue healing in various surgical defects. PRP derives from the centrifugation of peripheral blood and has a high concentration of growth factors that promote healing. Recently, the use of adipose-derived mesenchymal stem cells (ADMSCs) has been thoroughly investigated as a form of wound healing enhancement. ADMSCs are autologous stem cells deriving from fat tissue, with a capability of differentiation in specific cells, depending on the micro-environment that they are exposed to. The aim of the present comprehensive review is to record the experimental studies that have been published and investigate the synergistic use of PRP and ADMSC in animal models. The technical aspects of experimentations, as well as the major results of each study, are discussed. In addition, the limited clinical studies including humans are also reported. Future perspectives are discussed, along with the limitations of current studies on the long-term follow up needed on efficacy and safety.

## 1. Introduction

Wound healing is one of the most researched basic mammal functions [1]. It is the physiological process of maintaining tissue integrity in cases of trauma or any type of defect. As it is widely known, wound healing is described as four distinct but consecutive phases; haemostasis, the inflammatory phase, the proliferative phase, and the remodelling phase [1,2,3]. As these phases have been widely understood, various experiments have been conducted on regulating wound healing to lead to a better outcome.

Local or systemic factors can impair wound healing. A major local factor is the infection of a surgical wound. Systemic factors known for wound complications can be diabetes mellitus, smoking, obesity, cardiovascular disease, nutritional status, or syndromes affecting soft or hard tissue repair [2]. Treatment modalities that have been used to aid wound healing in such patients with impaired healing mechanisms vary from growth factors and hyperbaric oxygen to lasers, ultrasound, and negative pressure therapy [2]. These may have different indications depending on the type of target tissue and the defect trying to heal.

Growth factors are a well-established tissue healing promoter. It has been established since the 1970s that centrifuged plasma containing platelets has a high percentage of growth factors and secretory proteins [4]. It is now known if platelet-rich plasma (PRP) has a significant effect on all the phases of tissue healing, as the growth factors that it contains participate in all of these [5].

Recently, the use of mesenchymal stem cells has been added to the spectrum of regenerative medicine. After their first description in 2001 by Zuk et al. [6], the easy isolation of mesenchymal stem cells from adipose tissue has led to even more research on their healing effect, which is comparable to the cells deriving from the bone marrow [7].

This review aims to present the basic characteristics of platelet-rich plasma, adipose-derived mesenchymal stem cells and their synergistic effects on experimental models published in the literature.

## 2. Platelet-Rich Plasma

Platelet-rich plasma (PRP) has been introduced in clinical practice since 1970, as it was known for promoting healing due to the concentrated growth factors that were included. Since then, it has been extensively researched and it is now known that PRP can adequately promote tissue healing, remodelling, a decrease in inflammation, and, in general, the alteration of the local tissue environment [8].

PPR is autologous, as it is produced by the patient’s blood. After peripheral blood initial centrifuge, the upper layer is transferred to a vial and is centrifuged again and the lower level of the centrifuge product contains pellets of PRP [9]. The final product does not contain any red blood cells. The final PRP can be activated using calcium or thrombin, to accelerate growth factor excretion.

In vitro cellular cultures have shown that PRP can increase the proliferation rate when its concentration is of at least 300,000 platelets/mL. The ideal concentration for maximum cellular proliferation has been calculated at 1,500,000 platelets/mL. Any concentration higher than that does not seem to have a significant effect on the proliferation process [10].

PRP acts via the degranulation of a-granules, which contain growth factors such as platelet-derived growth factor (PDGF), epidermal growth factor (EGF), transforming growth factor (TGF), insulin growth factor 1 and 2 (IGF-1, IGF-2), vascular endothelial growth factor (VEGF), interleukins, and others [11]. Various studies have been implemented to assess the quantity of these factors in PRP. The concentration of PDGF, TGF, EGF, IGF in PRP compared to peripheral blood has been calculated as higher by 440.6, 346,6, 460, and 190%, accordingly [12,13]. The main characteristics of these factors are summarised in Table 1.

Platelets excrete 70% of their growth factors within 10 min of their activation and can climb up to 100% within the first hour. Afterwards, they can continue the production and excretion of growth factors up to 8 days, until the time they are destroyed. Therefore, the activation of PRP should be conducted at the time of usage and not earlier. After the destruction of the platelets, the macrophages that migrate to the defect area through the course of newly formed vessels (angiogenesis) excrete more growth factors as a response to the initial signal, and as a result, they accelerate the healing process [14,15].

PRP has been the subject of extensive research in the medical fields of orthopaedics, plastic surgery, and maxillofacial surgery, where it has multiple clinical applications [16]. Recent research focuses on neural regeneration and chronic pain [17]. Despite the massive amount of research, the treatment protocols that are proposed in various studies lack a common ground on study designs and PRP production processes, thus leading to a difficulty of comparing various therapeutic results [18,19]. Systematic reviews and meta-analyses on the efficacy and safety of PRP in clinical practice have concluded that they are effective without adverse effects on patients [20,21].

## 3. Adipose-Derived Mesenchymal Stem Cells

Adipose-derived mesenchymal stem cells (ADMSCs) are a heterogeneous population of cells that can be isolated and proliferated in vitro and can act as fibroblasts [22]. They can theoretically differentiate into any type of tissue, including bones, cartilage, or adipose tissue [6,23,24]. ADMSCs have a varying expression of hematopoietic growth factors. Typically, they do not express cell surface markers, such as cluster of differentiation (CD) 45, CD34, CD14, or even CD40, CD80, and CD86. Varying levels of CD44, CD90, CD71, GD2, and CD271 have been recorded. The positive expression of CD29, CD44, CD73, CD90, and CD105 has been recorded [25,26]. ADMSCs induce a local effect via the response to growth factors and anti-inflammatory proteins in response to cellular destruction and inflammation. These include prostaglandin-2, transforming growth factor b1 (TGF-b1), vascular endothelial growth factor (VEGF), and various interleukins (IL-4, IL-6, IL-10). These factors induce angiogenesis and tissue remodelling [27,28]. Furthermore, due to their immunogenicity, ADMSCs can inhibit the proliferation of inflammatory cells such as CD4+, CD8+ T-cells or natural killer cells [28].

Adipose tissue is present in all mammals and even in some non-mammals. Anatomically speaking, it is present in subcutaneous areas, intraperitoneally, and diffusely in any body part. Adipose tissue derives from the mesoderm, as do the skin, bones, cartilages, and hematopoietic cells also [29]. Lipogenesis includes the proliferation of preadipocytes, which later differentiate into mature lipocytes. This process is closely regulated by neuronic, hormonal, and paracrine pathways [30].

Mesenchymal stem cells can be easily collected by subcutaneous adipose tissue, as the latter is in abundance in mammals, in a high concentration, much higher than bone marrow-derived mesenchymal stem cells [31]. ADMSCs have high cellular activity, and can be easily used in various experimental models [32]. As they proliferate, they retain an original pool of ADMSCs in a non-differentiated status [33]. In addition, they have increased immunosuppressive properties and reduced immunogenicity, compared to other cell types, and thus can be widely used in vivo [34]. Their low immunogenicity is mainly due to the lack of expression of HLA class II [35]. ADMSCs qualify according to all the criteria set by the International Society for Cellular Therapy (ISCT) and the International Federation of Adipose Therapeutics and Sciences (IFATS) for use in clinical medicine [36,37]. A review published by Nolan et al. suggests that ADMSCs can provide good outcomes in healing, especially in compromised experimental models (e.g., diabetic rats) [38]. A certain parameter that must be considered is the type of storage and transportation protocol of ADMSCs, as any small change in temperature can diminish the potential benefit. In particular, an original study suggests that transportation and short-term storage can be an option for ADMSCs at −4 °C, but the optimal beneficial effects are present within the first 2 h after harvest [39].

## 4. Experimental Models

### 4.1. Periodontal Tissues

One of the first preclinical experimental models on the synergic effect of ADMSCs and PRP published was the one by Tobita et al. in 2008 [40]. They investigated the healing effects in the periodontal tissues of rats, by creating a defect on the alveolar bone of rats and implanting PRP only or ADMSCs and PRP. Histological investigation on timed periods after the implantation revealed greater tissue regeneration in the combined group, compared to the PRP group. The control group, with no implanted substances, had no tissue regeneration whatsoever. The researchers also hypothesised that ADMSCs may be able to differentiate to periodontal ligament cells and osteocytes, as these were present in the study group. The same research team repeated the experiments in a canine model in 2013 [41]. They used beagle dogs to examine the same hypothesis, concluding that after 2 months of implantation of the combination of ADMSCs and PRP in surgically created periodontal defects, the newly formed bone and the periodontal cementum were of significantly higher quality compared to the PRP and control group. Toward this orientation, Ding et al. examined if the combination of ADMSCs sheets and autologous platelet-rich fibrin (PRF) application is superior to their individual use in peri-implant mandibular bone defects of a canine experimental model. The group treated with the co-administration of ADMSCs sheets and PRF presented significantly increased new bone formation and re-osseointegration, as well as higher rates of bone remodelling activity, although all the treatment groups had formed larger quantities of new bone tissue compared to the control group. Additionally, the enhancement of the ASC sheets with PRF increased the proliferative and osteogenic capacity and led to significantly better results at the implant pushout test, offering implant stability. Thus, the combination of ASCs and PRF is endowed with multiple advantages regarding peri-implant tissue regeneration and stability [42].

An experimental work by Shafieian and colleagues [43] investigated in vitro the cell proliferation and differentiation of human ADMSCs after incubation with hydroxyapatite/tricalcium-phosphate (HA/TCP) and/or autologous PRP. Interestingly, a cell proliferation assay revealed that HA/TCP granules significantly reduced the proliferation rate and osteogenic differentiation ability of hADMSCs, but the co-application of PRP and HA/TCP scaffold enhanced these properties. Furthermore, HA/TCP decreased the mineralised nodule formation capacity, which was reversed by PRP. The application of PRP only led to significantly higher amounts of mineralised nodule formation. In vivo, the regenerative role of the aforementioned substances was examined in a canine model with 20 iatrogenic mandibular defects. The investigators formed four groups as follows: (I) autologous crushed bone graft, (II) no filling substance, (III) HA/TCP and PRP, and (IV) PRP-enriched hADMSCs in HA/TCP scaffold. Eight weeks later, groups III and IV presented statistically significant bone formation compared to group II, with sufficient incorporation into the original bone tissue. However, autologous crushed bone showed the best healing outcome, macroscopically. Concluding, the use of HA/TCP scaffold and PRP to augment hADMSCs effect significantly improved mandibular defect healing.

### 4.2. Tendons

In addition, the research group of Uysal et al. investigated the healing capacity of ADMSCs, mixed with PRP in the Achilles tendons of rabbits [44]. Surgical defects were made on both the tendons of rabbits and, during repair, a mixture of ADMSCs/PRP or PRP only (control) was used. The main results of this study were that, in the combined group, the strength of the tendons in 4 weeks was significantly higher, whereas a higher formation of collagen type I was seen, along with a higher expression of the fibroblast growth factor and vascular endothelial growth factor expression. Another interesting remark is that 11.53% of tenocytes in the experimental group were differentiated from ADMSCs, since the latter were stained with a fluorescent stain.

The effect of the combined treatment on tendon defects has been further studied. The tendinopathy of the supraspinatus was analysed retrospectively in 55 canines, which received a mixture of ADMSCs and PRP, and gait was evaluated in 90 days [45]. The canines evaluated presented a significant improvement in the total pressure index percentage, which represents the pressure the canine exerts on every paw step on the affected limb. In addition, an ultrasound examination revealed a reduction in the affected tendon in all the canines.

### 4.3. Osteoarthritis

Osteoarthritis is a medical condition widely targeted by regenerative medicine. Cartilage regeneration was evaluated in 2013 by Phuc et al. [46], who treated a surgical defect of the articular cartilage of mice hindlimbs by administrating either human ADMSCs (hADMCs) previously activated in vitro with PRP, or hADMSCs only. The mice were monitored for the recovery of hindlimb movement, and the researchers concluded that the study group of ADMSCs/PRP had a significantly faster recovery compared to the ADMSCs only and control groups. In addition, histopathology revealed higher cartilage regeneration by 45% in combined group, whereas the ADMSCs group showed a 30% increase and the control group a respective 20%increase after 45 days.

More osteoarthritis models have been published over the past years. Yun et al. [47] investigated the synergic effect on surgically induced knee ligament defects on canines. The canines, after a post-surgical period of soft tissue healing, were administered weekly injections of PRP, ADMSCs, or their combination for a month. After a 2-month period, the canines were sacrificed. The lameness score was measured before sacrifice, as well as extracellular matrix composition and inflammatory response histologically. In all the parameters, the combined group had a favourable outcome. In 2016, Hermeto et al. [48] induced osteoarthritis in rabbits and evaluated the healing with PRP, ADMSCs, and their combined use. ASMSCs were used as undifferentiated or differentiated into chondrocytes or osteocytes. They concluded that there was significantly improved regeneration in both ADMSCs groups, without a difference between them. Hsu et al. executed a similar experiment in 2018, concluding that a significantly better histological image was recorded in the combined group compared to the control group [49].

Ahmad et al. in 2020 [50] published an experimental study on rats comparing the efficacy of PRP, ADMSCs, and their combination on a surgically induced osteoarthritic knee. They concluded that interleukin-6 expression is significantly reduced in the combined group, with a significantly higher proteoglycan content compared to the other groups. In addition, a molecular cell proliferation marker, PCNA, is expressed at higher levels in the combined group.

### 4.4. Bone

The great need for bone grafts in the fields of neurosurgery, orthopaedic, and maxillofacial surgery urged research toward the investigation of substitutes with osteogenic properties. In the field of oral and maxillofacial surgery, the literature contains experimental reports of bone marrow-derived stem cells administration with promising results regarding bone restoration, while there are also clinical trials revealing clinical improvement after autologous bone marrow-derived stem cells and PRP injections. Janus et al. [51] studied the effect of human adipose stem cells (hADSCs), PRP, and collagen and their combination 38 days after iatrogenic mandibular osteoradionecrosis in rat models. After 28 days, micro-CT analysis revealed a statistically significant increase in bone volume following the administration of ADMSCs only (36%, *p* < 0.01) or in combination with PRP and collagen (19%, *p* < 0.05). PRP and collagen tended also to increase the bone volume, without achieving a statistically significant difference (*p* = 0.09). PRP and collagen administration led to a significant increase in the osteoblast levels, contrary to ADMSCs, while all the treatment groups had a significant reduction in the osteoclast count. In addition, fibrosis formation was significantly increased in ADMSCs, PRP and collagen, and their combined groups. The aforementioned results are consistent with the previous literature, suggesting the important role of ADSCs and PRP in bone formation and regeneration.

A model of medication-related jaw osteonecrosis was studied in 2016 [52]. Rats were administered zoledronic acid, a known medication causing jaw osteonecrosis. After a certain period, dental extractions were performed and PRP, ADMSCs, or their combination were placed in the post-extraction sockets. A significantly lower rate of osteonecrosis was observed in both the ADMSCs and combined group, along with better bone remodelling.

Da Silva et al. [53] studied the potential regenerative role of autologous ADSCs with osteogenic differentiation embedded in a PRP scaffold, with or without the administration of papaverine (PPV), in rats with surgical parietal defect versus autologous parietal bone graft transplantation. The rats were euthanatized after 28 days. In 6.7% of the total animals, which all belonged to the PRP and ADSCs and papaverine groups, neither newly formed bone trabeculae (NBT) nor osteoblastic activity (OA) was noticed. However, in 88.9% of the animals treated with ADSCs, the formation of bone trabeculae was noticed, confirming their osteogenic potential. Although not statistically significant, the autograft groups had better levels of NBT than the treatment groups, while a better OA was noticed in the control group euthanatized on day 14 and in the ADSCs and PRP and PPV group euthanatized on day 14. No statistically significant difference was revealed between the cellular groups with or without PPV. Nevertheless, the authors underlined that longer observational periods could reveal better bone formation levels among cellular groups.

Trying to meet the need for effective bone regeneration materials and taking advantage of the differentiation and proliferation capacity of ADSCs, Tajima and colleagues [4] were the first to investigate the regenerative role of autologous ADSCs, PRP, and type I collagen gel in rat calvarial defects. During preparation, ADSCs were mixed either with PRP or type I collagen gel. The experimental animals were administered either a combination of ADSCs/PRP or ADSCs/collagen gel or PRP only or collagen gel only or phosphate buffer solution (PBS). Interestingly, the ELISA measurements demonstrated that growth factors expression was significantly higher in ADSCs cultured in 5% PRP than in those preconditioned in 10% foetal bovine serum (FBS). Four weeks after surgery, the formation of new bone with lacunae and fusion with the original bone was observed only in the ADSCs/PRP group, while other groups did not present fusion with the original bone, but inflammatory cell infiltration locally, suggestive of insufficient healing. Four weeks later, the ADSCs/PRP group presented increased bone thickness. In vivo differentiation of a portion of ADSCs was also recognised after a PRP culture. All the results suggest in favour of the tremendous potential of PRP in increasing the regenerative effect of ADSCs in bone lesions.

Based on the gratifying results of bone mesenchymal stem cells and autologous PRP on bone regeneration published by Ueda et al. [54] and the extending role of ADSCs, Nakano et al. [55] aimed to clarify the role of dedifferentiated adipocytes (DFATs), which are adipocyte-derived fibroblast-like cells with great proliferation and differentiation capacity, alone or in combination with activated PRP (aPRP) in regenerating iatrogenic bone defects in rat calvaria. Four groups were formed and studied for 4 weeks as follows: (1) no implant, (2) Gelatin Sponge (GS), (3) GS seeded with DFAT, and (4) GS seeded with DFAT and PRP. The results revealed that the GS and rDFATs and aPRP group presented a better bone-forming capacity and bone quality than the GS and rDFATs group. The fact that almost all the 9-millimeter size bone defects completely closed in the former group is promising. In addition, pathological examination revealed fibrosis osteoconduction from the native bone in the GS and rDFATs and aPRP group. A result revealed using a Vitro Differentiation Assay during osteogenic differentiation and must be highlighted is that the growth and the proliferation rate of DFAT tended to increase in proportion to the aPRP concentration, suggesting that the optimum concentration should be chosen taking into consideration the cell type examined. The authors concluded by recognising the advantageous role of GS and rDFATs and aPRP administration in bone regeneration but drew attention to further research of its long-term properties.

### 4.5. Skin and Soft Tissues

MSCs-related tissue engineering has also introduced a new era in the field of angiogenesis. Based on the regenerative properties of ADSCs, Chen and colleagues [56] used a human PRP solution of different concentrations (2.5, 5, 7.5, and 10%) as a culture medium of ADCs to examine their neoangiogenetic properties both in vitro and in a mouse ischemic hindlimbs model. Compared to FBS culture (control), PRP-preconditioned ADSCs presented endothelial cell characteristics in vitro and significantly promoted neoangiogenesis, as the expression levels of angiogenic-related growth factors also reveal. Optimal outcomes were observed in 5 and 7.5% concentrations of PRP, resulting in better perfusion rates. ADSCs cultured in 5 and 7.5% were subsequently used in vivo and were compared with ADSCs cultured in 10% FBS, human umbilical vein endothelial cells (HUVECs), and phosphate buffer solution (PBS) only. The above were injected intramuscularly and serial laser Doppler blood flow meter measurements followed. On day 18, the PRP and HUVEC groups presented significantly higher revascularisation levels compared to the PBS and FBS groups. No significant difference was noticed between the PRP and HUVEC groups regarding perfusion ratios. As the laser Doppler images testify, the pre-enhancement of ADSCs with PRP significantly increased tissue perfusion in comparison to the FBS, HUVEC, and PBS groups. This is verified by histological examination, showing significantly higher capillary densities in PRP groups compared to the resting groups. The highest capillary density and angiogenesis effect was noted in the group with ADSCs preconditioned in 7.5% PRP. In conclusion, 5 and 7.5% PRP present promising properties as a substitute culture medium, enhancing the angiogenic role of ADSCs.

The effect of the combination of PRP and ADSCs is gaining interest regarding skin wound and thermal injury healing in the global literature. Early in 2009, Blanton et al. [57] evaluated the effect of autologous ADSCs, when administered either alone or in platelet-rich or platelet-poor fibrin gels (PRF), to promote full-thickness porcine lesions healing. The data obtained revealed that there was no significant difference in the re-epithelialisation rate, while the ADSCs and PRP group presented a significantly higher microvessel density compared to all the other groups, including ADSCs and PRF, a result indicative of the synergic effect of ADSCs and PRP. Moreover, the authors verified that in the ADSCs and PRP group, wound healing was characterised by significantly better cosmetic results compared to the other groups, which is one of the main goals in wound healing tissue engineering. Complete epithelial covering was achieved in the former group by day 16 to 21. Interestingly, the expression of vascular endothelial growth factors was significantly higher (approximately 7-fold) on the groups administered ADSCs. By way of conclusion, the authors strictly believe that a fibrin gel vehicle or scaffold containing growth factors is necessary for achieving wound healing, rendering ADSCs and PRP gel a promising totally autologous healing treatment agent.

Aiming at overwhelming the limited survival of stromal cells due to the hypovascular microenvironment of wound beds, Bhang et al. investigated the healing properties of human PRP, hADSCs, and their combination, all administered through a heparin-conjugated fibrin delivery system intraperitoneally [58]. The experimental animals were female mice, who underwent surgically full-thickness wounds on their back. After 16 days of treatment, the combined group presented better skin regeneration, since the wound edge distance, intersubcutaneous distance, and interpanniculus carnosus distance were significantly reduced compared to the other groups. Immunohistochemistry revealed a significant difference in the combined group at 16 days regarding epidermis and basal layer regeneration, while the coadministration of human PRP resulted in better hADSCs survival and proliferation on the third day and ameliorated regeneration through the enhancement of angiogenic paracrine factors and local angiogenesis, compared to PRP or hADSCs only.

Samberg et al., based on studies revealing the healing properties of ADSCs and PRP in full-thickness porcine wounds, as well as the biochemical and morphological properties of polyethylene glycol (PEG) [59], hypothesised that a PEGylated human PRP hydrogel enriched with hADSCs could lead to better healing results through increased growth factor levels and neoangiogenesis [60]. Fourteen days after incubation, the in vitro results showed that the ASC proliferation and growth factor gene expression were increased in higher concentrations of PRP. An in vivo study in a rat model with iatrogenic skin lesions included the following treatment: (1) 250 mL of saline (control), (2) platelet free plasma (PRF) gel, (3) PFP and ASCs gel, (4) PRP gel, and (5) PRP and ASCs gel. PRP and ASC hydrogels showed significantly earlier and denser vessel presence 8 days after the surgery, compared to the control group, cell or plasma treatment only. The former presented a lower granulation tissue level, while all the treatment groups presented similar re-epithelialisation rates and epidermis thicknesses. Therefore, the co-administration of ADSCs and PRP offered a synergic effect regarding the re-vascularisation of skin lesions, even though the present study failed to prove statistically significant differences regarding other healing parameters, such as re-epithelisation, maybe due to the study’s technical limitations.

In 2018, Mansoub et al. studied the healing potential of autologous PRP or autologous keratinocyte-like cells (KLCs) derived from ADMSCs and their combination in streptozocin-induced diabetic rats [61]. After inducing second-degree burn injuries and the subcutaneous administration of KLCs and/or PRP, wound measurements were performed, and biopsies were taken on the 3rd, 7th, 10th, and 14th days. On the 14th day, the wound contraction ratio, as a quantitative measurement of wound closure, was significantly higher in the combined group compared to the control group or the groups treated with KLCs or PRP alone. In addition, the mRNA measurement of different healing markers indicated an elevated expression in the PRP group, both on days 7 and 14, compared with the KLC and control group, while the highest expression was noted in the PRP and KLC group, suggesting the best tissue response. Collagen-1 formation was found to be promoted in the PRP and combined group in different stages, while in the KLC group, significant collagen formation was noticed after day seven. In all the treatment groups, a statistically significant decrease in inflammation was noted, notably in the combined group. The researchers concluded that wound healing was more prominent in the combined group, since PRP promotes the healing effect of KLCs.

Based on the expanding global need for efficient diabetic lesions wound-healing strategies, Ebrahim and colleagues also used the abovementioned experimental model of streptozocin-induced diabetic rats to examine the effect of individual or combined autologous PRP and ADSCs treatment [62]. An addition to the previous studies, however, is that the authors also elucidate the role of the Notch signalling pathway in healing, given its key role in cellular processes disrupted in diabetic wound homeostasis. After 14 days, almost complete healing with sufficient filling of wound periphery, granulation tissue formation, epithelisation, and minimal signs of inflammation were noted in the shame (non-diabetic, no-treatment group) and PRP-ADSCs groups, followed by the ADSCs group, while the PRP-treated wounds had the least healing noted among the treatment groups. Of note are also the results revealing the downregulation of Notch1 pathway-related genes to almost normal levels in the diabetic rats treated with combined ADSCs and PRP. This suggestion demonstrates that Notch1 pathway inhibition in diabetic lesions leads to increased EPSC proliferation and angiogenesis, rendering it a future therapeutic target.

The potential regenerative properties of PRP combined with ADSCs have also aroused interest regarding their use in reconstructive surgery, where the current use of grafts and flaps is still compromised by issues of viability and flexibility. In 2015, Seyhan et al., administrating autologous PRP, ADSCs, and their combination in a rat experimental model of autologous fat graft transplantation in the scalp, concluded that the combined administration of PRP and ADSCs resulted in weight and volume maintenance of the graft, compared to the other groups [63]. In addition, according to histological examination, the combined treatment group presented a significantly higher percentage of blood vessels and viable adipocytes, with lower cyst and fibrosis formation, when compared to the control group. The above is confirmed by the ELISA results, showing significantly higher levels of VEGF, FGF, and TGF-β in the combined group, and thus, highlighting the synergic effect of PRP and ADSCs for improving fat graft properties. Similarly, Gao et al. studied the local administration of autologous ADSCs embedded in autologous PRP scaffold under a full-thickness skin graft [64]. The results suggest that PRP gel enhanced ADSCs survival in the wound bed in the combined group, while it decreased the elastic modulus, increasing the flexibility of the tissue. A histological examination also revealed significant differences between the PRP and ADSCs and the control group, with increased epidermis thickness and ameliorated collagen arrangement in the combined group. The latter group, based on the results of laser perfusion imaging and immunohistochemistry, presented increased blood flow under the graft and enhanced early-stage neovascularisation, probably through increased vascularisation factors and cytokines secretion. The authors also confirm that isolated ADSCs retain the differentiation potential of stem cells, toward either the osteogenic or adipogenic group in the present study. In conclusion, ADSCs in PRP scaffolds promise gratifying results regarding skin grafts survival and neovascularisation.

### 4.6. Cardiovascular Tissue

The literature contains limited experimental studies regarding the applications of MSCs and PRP in the regeneration of cardiovascular tissue, despite their proven safety and regenerative properties. In 2016, Mörschbächer et al. investigated the effect of autologous PRP, adipose MSCs, and the combination of adipose MSCs in PRP scaffold in rabbits with doxorubicin-induced dilated cardiomyopathy [65]. The measurements of serum troponin-I were highest in PRP, followed by the control group, combined group and lastly, by the MSC group. Electrocardiography revealed no statistically significant difference among groups, while echocardiography revealed increased ejection and shortening fraction values in the MSCs group. The histological examination was suggestive of more lesions in PRP group, while the rabbits of the MSC group had the best histological outcomes. In summary, the authors highlight that while the MSCs autologous transplant was of benefit for the rabbits with DCM, the administration of PRP, either alone or in combination with MSCs, worsened the functional and histological profile of heart function and offset the beneficial role of MSCs.

### 4.7. Neural Tissue

Although the literature contains numerous reports of mesenchymal stem-cells use for neural repair and regeneration strategies, there is a lack of evidence regarding the synergic role of ADMSCs and PRP in neural trauma and degeneration. To meet this need, Salarinia et al. proposed and studied the effect of autologous ADMSCs, PRP, and their co-administration in a rat experimental model one week after iatrogenic spinal cord injury [66]. The results revealed a statistically significant increase in the Bax gene in the ADMSCs and combined group and of the Bcl-2 in the combined group. Caspase 3 gene expression and cell apoptotic index were decreased in all the treatment groups, but mainly in the combined group. The outcomes reflecting axon regeneration were also favourable toward the combined group, while all the treatment groups led to improved hindlimb motor function after the fifth week. The study highlighted the advantageous properties of the co-administration of autologous AD-MSCs and PRP in neural tissue lesions and degenerative conditions.

All the experimental studies are summarised in Table 2.

## 5. Applications in Clinical Medicine

As expected, very few studies have been conducted in clinical medicine. Orthopaedics is a major area investigating the use of ADMSCs and PRP, mainly in arthroscopy or local injections. The related studies are mainly case series and do not investigate possible safety concerns of these products on humans. In addition, no standardised protocols have been established. Thus, further experimental investigation is needed before establishing this treatment in humans [67].

A recent network meta-analysis comparing the use of ADMSCs with PRP, among others, in the management of knee osteoarthritis, concludes that PRP provides patients with the most effective functional outcome, whereas the use of mesenchymal cells has the greatest outcome on pain management. Of the studies included in this meta-analysis, only three used ADMSCs and they were not compared directly with PRP. In addition, none of the included studies examined the combined use of mesenchymal cells and PRP [68].

Another systematic review on the use of PRP and ADMSCs in dermal wound healing states that both are safe in human use and have superior results compared to traditional techniques, but more randomised trials are needed to verify these results [69]. As with the aforementioned research on osteoarthritis, no studies are included that combine the use of the two autologous substances.

A recent clinical study on humans reports that the combined use of PRP and ADMSCs injected in the scalp of patients with androgenic alopecia provides a time-related increased hair density, but there was no control group [70].

Another clinical study reports the use of ADMSCs and PRP in surgical treatment for Crohn’s disease refractory fistulas [71]. The long-term follow up revealed a significant clinical improvement with no adverse effects.

The applications in clinical medicine are summarised in Table 3.

## 6. Conclusions and Future Perspectives

Wound healing and tissue regeneration have been a major issue in clinical medicine for the past few decades. Their management remains a challenge since local factors or systemic comorbidities impair healing mechanisms. As per all the recent research on the use of ADMSCs and PRP, their combined use can offer precedence in the equilibrium between tissue healing and healing impairment. As both are autologous, it can be safely said that the human organism has a reservoir of concentrated healing aids that can be focused on a certain area and provide the acceleration of a better healing process. As depicted from the published preclinical studies, there is a need for a standardised protocol on the in vitro preparation of both ADMSCs and PRP, as various techniques have been proposed and no studies are comparing the differences in efficacy. In addition, there is no knowledge of their long-term effects.

Skin wounds, osseous defects, tendinopathy, and chondropathy are the most researched areas of tissue regeneration with the use of PRP and ADMSCs combination. Nevertheless, new areas of wound healing can be assessed. For example, hernia repair could be enhanced using these substances. There is only one publication concerning ventral hernias, but bone marrow mesenchymal stem cells were used [72]. As adipose tissue is more easily accessible, especially during hernia repair, this modification could provide a better clinical modality. In addition, primary findings in the use of PRP in vascularised free flaps for major reconstruction have shown an improvement in tissue regeneration [73]. If the long-term safety of the ADMSC is established, especially the effect on tumour formation, their clinical use could also be implemented into microsurgery.

Most of the studies included in this comprehensive review suffer from the same drawbacks. The long-term outcomes are not widely discussed, small sample sizes are used in the clinical models, and the PRP and ADMSC concentrations due to different protocols are not always comparable between the various studies. These limitations cannot provide a basis for a further analysis of the results that the researchers suggest.

In conclusion, the synergistic effect of PRP and ADMSC provides a well-established promoter in tissue healing. Further preclinical studies to assess the long-term outcomes, as well as the regeneration of other tissues, along with well-designed clinical studies, are needed to establish this technique in clinical medicine.

## Figures and Tables

**Table 1:** Main growth factors deriving from platelets and their functions.

Growth Factor	Main Functions
PDGF	Proliferation of mesenchymal cells Chemotaxis of granulocytes, monocytes, and fibroblasts Control of extracellular matrix Positive impact on bone formation If in excess, negative impact on bone formation
TGF	Control of cellular mitosis and differentiation Paracrine action on mesenchymal cells, inducing proliferation and differentiation
EGF	Positive impact on cellular differentiation Induction of proliferation and differentiation of mesenchymal cells Induction of excretion of other growth factors
IGF-1	Proliferation of osteoblasts
VEGF	Induction of angiogenesis

**Table 2:** Experimental studies on the combined use of PRP and ADMSC.

Expermental Study	Type of Tissue	Animal Model	Time Frame	Main Result	Dosage	Route	ASC/PRP Sample Size
Tobita et al., 2008 [40]	Periodontal tissue	Rats	2, 4, 8 weeks	Evident bone regeneration compared to PRP only or to no-implementation	1 × 10^7^ A–SCs/mL, 1 mL of PRP	Locally	24
Blanton et al., 2009 [57]	Skin	Pigs	3, 7, 14, 21 days	No significant difference in the re-epithelialisation rate ADSCs + PRP group presented increased microvessel density and VEGF level ADSCs + PRP group presented significantly better cosmesis	1.8 × 10^6^ A–MSCs, 3 mL of PRP	Locally	3 pigs/44 wounds per pig/8 wounds per treatment
Uysal et al., 2012 [44]	Achilles tendon	Rabbits	4 weeks	Significant increase in tendon strength, collagen formation, fibroblast growth factor, and VEGF expression	10 × 10^6^ ADMSCs, PRP gel	Locally	6
Bhang et al., 2013 [58]	Skin	Mice	16 days	Prominent skin regeneration, increased angiogenesis and hASCs proliferation in the combined group Increased survival of the hASCs when combined with PRP	hASCs (1.8 × 10^6^ cells per defect)-μL of human PRP mixed with HCF	Intraperitoneally	6
Tobita et al., 2013 [41]	Periodontal tissue	Canine	4, 8 weeks	Significant increase in bone formation in 8 weeks compared to PRP only or to no-implementation	0.3 mL of PRP and/or KLCs (4 × 10^5^ cells in 0.3 mL culture medium)	Locally	8
Phuc et al., 2013 [46]	Articular cartilage	Mice	45 days	Significantly faster hindlimb movement and higher cartilage regeneration in the combined group	2 × 10^6^ ADSCs, 200 μL of PRP	Intra-articular	4
Seyhan et al., 2015 [63]	Fat	Rats	12 weeks	Combined group presented highest mean weight and volume of fat grafts, increased survival of adipocytes, and increased angiogenesis and levels of growth factors	0.2 mL 5 × 10^6^ ADSCs, 0.2 mL of PRP	Local, subcutaneous	10
Mörschbächer et al., 2015 [65]	Myocardium	Rabbits	15 days	MSC group presented improved heart function Combined group presented DCM deterioration	106 cells MSCs, 1 mL of PRP	Intracardiac	5
Tajima et al., 2015 [4]	Bone	Rats	4, 8 weeks	PRP significantly increased levels of growth factors secreted by ADSCs Combined group presented formation of thick new bone in fusion with the original	2 × 10^5^ ADSCs, 20 µL of PRP	Subperiosteally	10
Canapp et al., 2016 [45]	Supraspinatus tendinopathy	Canines	90 days	Significant improvement in limb movement, reduction in inflammation in combined group	5 × 10^6^ ADMSC, PRP gel	Locally	55
Barba-Recreo et al., 2016 [52]	Alveolar bone	Rats	7 days	The ADMSC groups showed higher bone remodelling	1 × 10^6^ ADMSCs, 25 mL of PRP	Locally	13
Hermeto et al., 2016 [48]	Articular cartilage in knees	Rabbits	60 days	Improved tissue regeneration in both AMDSCs groups	4 × 10^6^ undifferentiated or differentiated ADMSCs, 0.5 mL of PRP	Intra-articularly	6
Yun et al., 2016 [47]	Cranial cruciate ligament	Canines	3 months	Increased mobility and compression strength of articular surfaces, increased collagen deposition, and significantly lower inflammation response in the combined group	1 × 10^6^ ADMSCs, 1 mL of PRP	Locally	6
Shafieian et al., 2017 [43]	Μandibular bone	Canines	8 weeks	In vitro, HA/TCP granules significantly decreased proliferation and osteogenic differentiation ability of hAdMSCs, while PRP enhanced these properties In vivo, PRP-enriched hAdMSCs embedded in HA/TCP granules led to significant bone formation, with incorporation into original bone	5 × 10^4^ cells hAdMSCs-HA/TCP granules (30 µg/mL), PRP 20%	Locally	5
Janus et al., 2017 [51]	Μandibular bone	Rats	28 days	ADSCs, as well as their combination with PRP/COL, increased bone volume ADSCs increased jaw region and fibrosis Osteoclast levels were significantly decreased in all experimental groups Injection of PRP/COL increased osteoblasts	1 million hADSCs in 1.0 mL of saline or 20% PRP 1, 10% collagen, with a 0.2-milliliter injection volume for every rat	Intramandibularly	5
Mansoub et al., 2018 [61]	Skin	Rats	3, 7, 10, and 14 days	Earlier wound contraction, increased levels of wound healing markers, and promoted collagen formation in PRP and combined group In all treatment groups, statistically significant decrease in inflammation, mainly in the combined group	4 × 10^5^ cells, 0.3 mL of PRP	Subcutaneously	6
Ding et al., 2018 [42]	Peri-implant bone	Canines	4, 8 weeks	Combined group presented significantly increased bone formation and re-osseointegration and the highest bone remodelling rate	Not specified	In the peri-implant area	9
Samberg et al., 2018 [60]	Skin	Rats	8, 12 days	In vitro, ASC proliferation and vascular growth factor gene expression were increased in proportion with PRP concentration In vivo, co-administration of PRP and ASC hydrogels led to increased angiogenesis by day 8 compared to controls	Not specified	Locally	4
Hsu et al., 2018 [49]	Osteochondral defects in knees	Rabbits	12 weeks	Higher extracellular matrix and cellularity of the combined group	1 × 10^6^ of ADSCs/0.2 mL suspended in 0.8 mL of platelet rich fibrin	Intra-articularly	6
Chen et al., 2018 [56]	Vascular	Mice	0, 1, 4, 7, 11, 14, and 18 days	PRP-preconditioned ADSCs groups presented higher levels of revascularisation and tissue perfusion rates compared to PBS and FBS groups 5 and 7.5% PRP constitute a promising culture medium	3 × 10^6^ preconditioned ADSCs	Intramuscularly	
Gao et al., 2019 [64]	Skin	Rat-Male	2, 4, 12 weeks for every subgroup	Isolated ADSCs retain the multidirectional differentiation potential of stem cells PRP scaffolds increased ADSCs concentration in skin grafts Combined group presented decreased elastic modulus, increased flexibility and skin thickness, improved collagen arrangement, increased skin neovascularisation	1 × 10^6^ cells in 1 mL of PRP	Subcutaneously	18
Da Silva et al., 2020 [53]	Bone	Rats	14 and 28 days	Autograft groups presented higher levels of neoformed bone trabeculae and osteoblastic activity	5 × 10^5^ cells/mL of ADSCs, 0.05 mL/sample of papaverine, no data for PRP	Locally	6
Salarinia et al., 2020 [66]	Spinal cord	Rats	3, 5 weeks	Combined use of PRP and AD-MSC offers better results limiting cell apoptosis and axonal degeneration and improving locomotor function compared to monotherapy or control group	2 × 10^5^ ADSCs, 10 µL of PRP	Locally	12
Nakano et al., 2020 [55]	Bone	Rats	4 weeks	Adipose-derived dedifferentiated fat (DFAT) cells combined with aPRP presented significantly increased cell proliferation	Not specified	Locally	5
Ahmad et al., 2020 [50]	Articular cartilage in knees	Rats	30 days	The combined group showed significant reduction in inflammation, significantly greater improvement in proteoglycan content, and increased cell proliferation and PCNA expression	30 μL PRP, 1 × 10^6^ per 100 μL of ADMSCs	Intra-articularly	10
Ebrahim et al., 2021 [62]	Skin	Rats	3,7, 10, and 14 days	Combined administration of PRP + ADSCs presented better healing results, since re-epithelialisation, granulation, and collagen-angiogenesis and increased epidermal thickness were noticed In combined group, downregulation of Notch signalling as well as increased epidermal stem cells (EPSCs) were noted	2 × 10^6^ ADSCs, 4 mL of PRP	Locally	14

**Table 3:** Applications of the combined use of ADMSC and PRP in clinical medicine.

Authors	Type of Study	Tissue—Disease	Number of Patients	Main Outcomes
Stevens et al., 2018 [70]	Prospective, observational pilot study	Skin—androgenetic alopecia	10 male patients with stage II and III androgenetic alopecia	Hair density increased significantly within 6 to 12 weeks after a single injection of stromal vascular fraction (SVF), which is rich in AD-MSCs in combination with platelet-rich plasma (PRP) In addition, it was noted that new terminal hairs have grown from existing follicular units, but also from previously inactive empty hair follicles
Wainstein et al., 2018 [71]	Prospective, observational pilot study	Skin—refractory perineal Crohn’s disease	9 patients, 11 fistulas	Combined therapy with AD-MSCs, PRP, and endorectal advancement flaps was studied 10/11 fistulas displayed complete healing, while 1/11 displayed partial healing No evidence of relapse or adverse complications were noted Perianal Disease Activity Index, showing severity of perianal disease, is decreased, while Inflammatory Bowel Disease Questionnaire-32, reflecting quality of life, improved postoperatively
